# Identifying potential biomarkers related to pre-term delivery by proteomic analysis of amniotic fluid

**DOI:** 10.1038/s41598-020-76748-1

**Published:** 2020-11-12

**Authors:** Subeen Hong, Ji Eun Lee, Yu Mi Kim, Yehyon Park, Ji-Woong Choi, Kyo Hoon Park

**Affiliations:** 1 Department of Obstetrics and Gynecology, College of Medicine, The Catholic University of Korea, Seoul, Korea; 2 Center for Theragnosis, Biomedical Research Institute, Korea Institute of Science and Technology, Seoul, Korea; 3 Department of Obstetrics and Gynecology, Seoul National University College of Medicine, Seoul National University Bundang Hospital, 82, Gumi-ro 173 Beon-gil, Bundang-gu, Seongnam, 463-707 Korea; 4 Wide River Institute of Immunology, Seoul National University, Hongcheon, Korea

**Keywords:** Immunology, Medical research, Pathogenesis

## Abstract

We sought to identify biomarkers in the amniotic fluid (AF) and specific signaling pathways related to spontaneous preterm delivery (SPTD, < 34 weeks) in women with preterm labor (PTL) without intra-uterine infection/inflammation (IUI). This was a retrospective cohort study of a total of 139 PTL women with singleton gestation (24 + 0 to 32 + 6 weeks) who underwent amniocentesis and who displayed no evidence of IUI. A nested case–control was conducted using pooled AF samples (n = 20) analyzed via label-free liquid chromatography-tandem mass spectrometry. In the total cohort, an ELISA validation study was performed for seven candidate proteins of interest. Proteomic analysis identified 77 differentially expressed proteins (DEPs, *P* < 0.05) in the AF from SPTD cases compared to term delivery controls. ELISA validation confirmed that women who had an SPTD before 34 weeks had significantly independently lower levels of VEGFR-1 and higher levels of lipocalin-2 and the Fc fragment of IgG binding protein in the AF. Five principle pathways associated with the 77 DEPs were identified, including glycolysis, gluconeogenesis, and iron homeostasis. The proteomic analysis data of AFs from women with PTL identified several novel biomarkers and specific protein pathways related to SPTD in the absence of IUI.

## Introduction

Preterm labor with intact membrane (PTL) complicates 2–3% of pregnancies and accounts for approximately one-third of preterm births, causing significant neonatal mortality and morbidity^1,2^. PTL is regarded as a complex syndrome with multiple etiologies that include genetic predisposition, infection and/or inflammation, decidual hemorrhage and vascular disease, hormonal dysregulation, and uterine overdistension^3^. To date, the only PTL mechanism that has been causally linked to spontaneous preterm delivery (SPTD) is infection/inflammation^4^, whereas the others are mostly implicated by associations suggested by epidemiologic, clinical, experimental, or placental pathologic observations^3^. Therefore, identifying biomarkers for biochemical changes that may be causally linked to SPTD in cases other than infection/inflammation is important for implementing mechanism-based treatment and prevention of PTL followed by SPTD.

While approximately 40% of SPTD cases are associated with mostly subclinical inflammations/infections, more than half of the SPTD cases are of unknown etiology (idiopathic, meaning that no identifiable cause is found)^5^. Despite the clinical relevance of idiopathic PTL for considering subfractions of prevalence, this area of study has received little attention from investigators involved in biomarker research mainly because the definition of idiopathic PTL subgroups using objective criteria has not yet been determined. Few studies have been carried out to explore single or a few biomarkers as potential etiologic agents of idiopathic preterm labor and delivery. They revealed that potential biomarkers involved in idiopathic preterm labor and delivery included five SELDI peaks in amniotic fluid (AF) Q-profile, placenta villous hypermaturation and senescence-associated β-galactosidase activity in the placenta, and protein Z levels and FokI Vitamin D receptor polymorphism in maternal blood^6–9^. However, these studies have limited their analyses to specific target proteins or to those identified from single mass peaks, thereby not reflecting the total of proteins involved in multiple and intricate pathways for idiopathic preterm labor and delivery. Moreover, idiopathic preterm labor and delivery was usually defined using either AF or placental analysis alone, which may have led to the inclusion of many infection/inflammatory cases in their analyses.

The AF proteomics-based approach is ideally suitable for the study of complex syndromes such as idiopathic preterm labor and delivery because it allows simultaneous evaluation of multiple protein signatures, providing new insight about the cause of disease^10^. The purposes of this study were to comprehensively identify AF biomarkers related to SPTD in the absence of intra-uterine infection/inflammation (IUI) in women with PTL using label-free liquid chromatography-tandem mass spectrometry (LC-MS/MS), and to explore specific signaling pathways that are activated in these cases.

## Methods

### Study design and participants

This retrospective cohort study was approved by the local ethics committee of Seoul National University Bundang Hospital, Seongnamsi, Republic of Korea (project number B-1105/128-102). Written informed consent for the collection and use of AF samples was obtained from all study subjects prior to the amniocentesis procedure. All methods were carried out in accordance with relevant guidelines and regulations of Seoul National University Bundang Hospital. All participants were recruited from Seoul National University Bundang Hospital between July/2004 and December/2017. Women with singleton pregnancies who were diagnosed with PTL at 24 + 0 to 32 + 6 weeks of gestation and who underwent amniocentesis were eligible for this study. The inclusion criteria were as follows: (1) live fetus; (2) AF samples available for analysis; (3) no evidence of clinical chorioamnionitis at admission or during hospitalization; (4) cervical dilatation ≤ 3 cm by digital examination; (5) absence of preterm premature rupture of membranes; and (6) absence of major congenital anomalies. We excluded patients with findings associated to intra-uterine infection/inflammation, which were defined as the presence of at least one of the following based on previous studies: elevated AF interleukin (IL)-6 levels (≥ 1.0 ng/mL) or white blood cell (WBC) counts (≥ 50 cells/mm^3^), positive AF culture or presence of histologic chorioamnionitis^11–15^, because the current study primarily focused on the non-infectious and non-inflammatory causes of SPTD. We used both the last menstrual period and a first or second trimester (≤ 20 weeks) ultrasound scan to determine gestational age. PTL was defined as regular uterine contractions at a frequency of five minutes or less with a cervical change (effacement, dilation or softening) that required hospitalization before 37 weeks of gestation.

In the discovery phase, a nested case–control study was conducted on 10 women who had SPTD before 34 weeks (case subjects), and 10 gestational-age matched women with term delivery (TD) (control subjects), randomly selected from a total cohort of 139 women with PTL in the absence of IUI who satisfied the inclusion/exclusion criteria. The AF proteome profiles of the case and control groups were compared using label-free quantitative mass spectrometry based on spectral counting. The primary and secondary outcome measures were SPTD before 34 weeks of gestation and SPTD within 14 days of sampling, respectively.

### Collection and storage of AF samples

A transabdominal amniocentesis was performed after consent, using an aseptic technique with ultrasound guidance for assessing AF for infection/inflammation and fetal lung maturity. Following previously described methods^16^, AF microbial cultures were performed for aerobic and anaerobic bacteria and genital mycoplasma (*Mycoplasma* *hominis* and *Ureaplasma* *urealyticum*), and a WBC count was performed. The remaining AF was centrifuged at 1500 *g* at 4 °C for 10 min and the supernatant was immediately stored in aliquots at − 70 °C until assayed. Medications such as tocolytics, corticosteroids or antibiotics were commenced after sampling.

To exclude subclinical intra-amniotic inflammation, IL-6 was assayed in the stored AF samples using the standardized enzyme-linked immunosorbent assay (ELISA) human IL-6 DuoSet Kit (R&D System, Minneapolis, MN, USA) in duplicates, according to the protocol recommended by the manufacturer. The intra- and inter-assay coefficients of variation (CV) were < 10%.

### Definition, diagnosis, and management of preterm labor

Management of PTL has been previously described in detail^16^, as well as in the Supplementary Material. The use and choice of a tocolytic drug, management of intra-amniotic infection/inflammation (IAI), and the decision of when to deliver a baby of a PTL patient were left to the discretion of attending physicians. Women with PTL were treated initially with hydration. If uterine contractions persisted**,** they received continuous intravenous tocolytic therapy with magnesium sulfate, ritodrine, or atosiban. At our institution, women with PTL were not given antibiotics to prolong pregnancy**,** except for development of clinical signs of chorioamnionitis and treatment of clinically diagnosed (or suspected) IAI. Maternal or fetal health status was carefully monitored for the development of clinical signs of chorioamnionitis and/or fetal compromise, both of which are indications for induction of labor. Acute histologic chorioamnionitis was diagnosed when acute inflammatory change was detected in any tissue sample (umbilical cord, chorionic plate, chorion-decidua, or amnion), in accordance with previously published criteria^17^. Clinical chorioamnionitis was diagnosed following the criteria proposed by Gibbs et al^18^.

### Mass spectral analysis of AF samples

Total protein concentration in AF samples was individually determined by a BCA assay (Micro BCA Protein Assay Kit, Thermo Fisher Scientific, Bremen, Germany). The pooled AF samples of SPTD case and TD control groups were generated by combining equal amounts of 10 individual AF samples from each group and further filtering them by centrifugation for 5 min at 16,000*g* and 4 °C. The 14 most abundant proteins were removed from 200 μg of pooled AF samples and subjected to tryptic digestion followed by high-pH reversed-phase fractionation as described in the Supplementary Material.

LC-MS/MS analyses of the fractionated peptide samples were performed using an online Thermo Easy nLC 1000 (Thermo Fisher Scientific, Bremen, Germany) system interfaced to a Thermo quadrupole-orbitrap Q-Exactive Mass Spectrometer (Thermo Fisher Scientific, Bremen, Germany), controlled by Xcalibur version 2.0.6 software (Thermo Fisher Scientific, San Jose, CA, USA) as described in the Supplementary Material.

### Protein identification and label-free quantitative analysis

The SEQUEST search algorithm (Sorcerer v 4.3.0 built, Sage-N Research, Milpitas, CA) was used for searching each LC-MS/MS file against the UniProt protein database (42,083 entries; released in Mar 2015). Scaffold 4 (version 4.3.2, Proteome Software Inc., Portland, OR) was used to filter peptide and protein identifications for an estimated false discovery rate (FDR) of less than 1%. Three replicate LC-MS/MS runs from each group of sample were performed and the spectral counts of all identified proteins were log_2_-transformed and compared using the R statistical programming with a power law global error model (PLGEM, https://www.bioconductor.org) in order to identify proteins showing statistical significant changes (*p*-value of 0.05) between the SPTD case and TD control groups^19–21^. The materials and methods used for complete proteomic analysis are described in detail in the Supplementary Material.

### Ingenuity pathway analysis (IPA)

Uniprot accession numbers of the DEPs and their corresponding log_2_ ratios between spectral counts from SPTD and TD groups were uploaded to the web-based Ingenuity Pathway Analysis (IPA) software (Version 26127183; QIAGEN, CA, USA) for functional analysis and protein interaction networks. These details were further described in the Supplementary Material.

### Enzyme-linked immunosorbent assay (ELISA)

To validate proteomic data on differential expression, we performed an immunoassay to determine AF levels of seven candidate proteins in a total cohort of 139 individual samples. The levels of insulin-like growth factor-binding protein-4 (IGFBP-4), lipocalin-2, S100 calcium binding protein A8 (S100A8), S100 calcium binding protein A8/A9 complex (S100A8/A9), vascular endothelial growth factor receptor 1 (VEGFR-1) (DuoSet ELISA; R&D Systems, Minneapolis, MN), carbonic anhydrase 1 (CA-1) (Human CA1 ELISA; Express Biotech International, Frederick, MD), and Fc fragment of IgG binding protein (FCGBP) (Immunotag Human FCGBP ELISA; G Bioscience, St. Louis, MO) were measured by ELISA kits used according to the manufacturers’ instructions. The ranges for the protein standard curves and their dilution ratios are described in detail in the Supplementary Material. The intra- and inter-assay CVs were < 10% for all the analyzed proteins, except for IGFBP-4 and S100A8/A9, for which the inter-assay CVs were 11.5% and 12.5%, respectively.

### Statistical analysis

All statistical analyses were performed with IBM SPSS 25.0 (IBM SPSS Inc., Chicago, IL). Continuous data were compared using the non-parametric Mann–Whitney *U*-test and categorical data were compared using Fisher’s exact test or χ^2^-test, as appropriate. Multivariate analyses were further performed to evaluate the independent association of the levels of AF candidate proteins which presented a *P* value < 0.1 in the univariate analysis, adjusting for baseline clinical parameters (i.e., parity and AF IL-6 levels). Interventions (i.e., administration of corticosteroids and antibiotics) were not included in the logistic regression model, because these were not baseline variables and were dependent on decisions made by the obstetricians. Two-sided *P* values < 0.05 were considered statistically significant.

## Results

**Figure 1:**
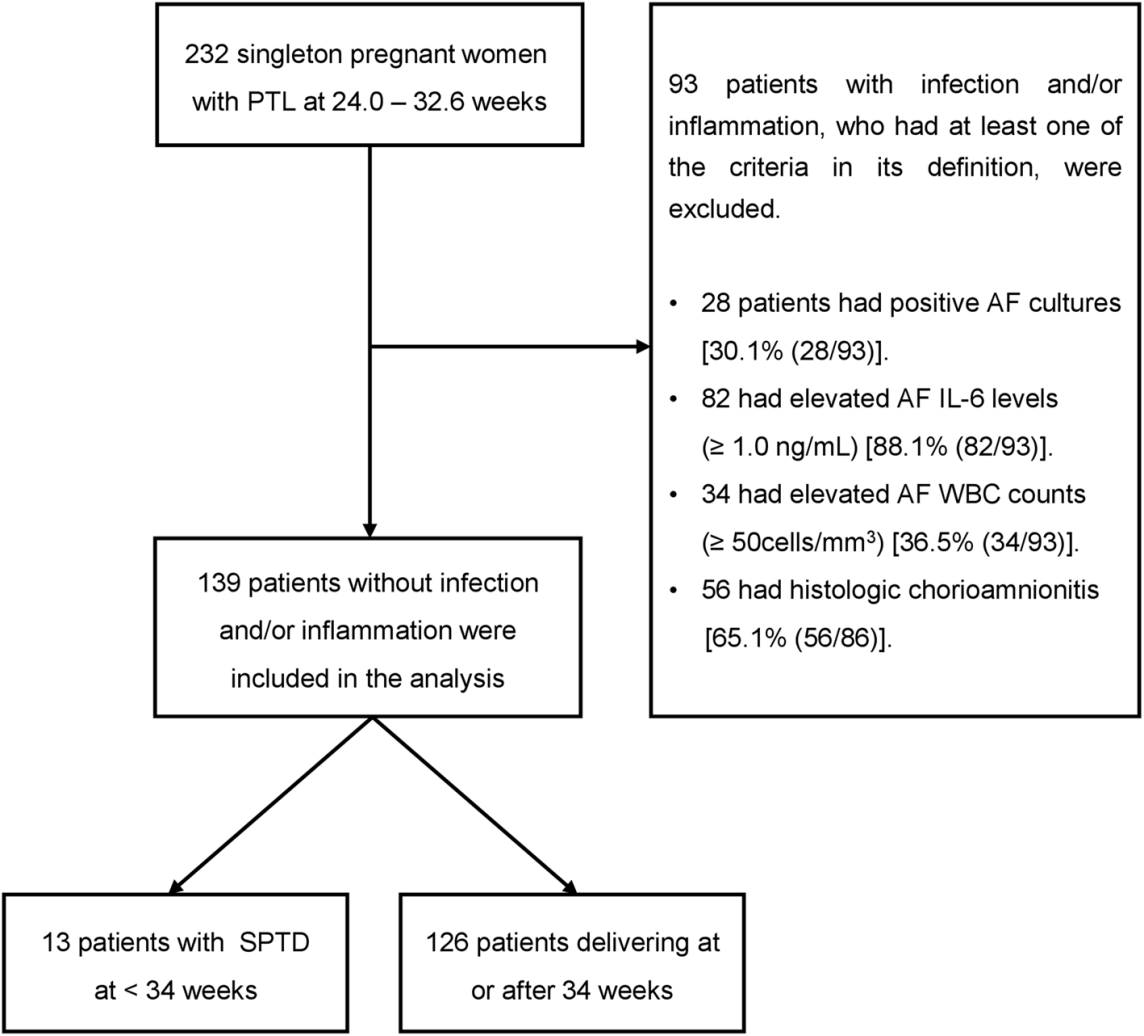
Flow-chart showing inclusion and exclusion of patients for the study.

During the study period, 232 women with PTL between 24 + 0 and 32 + 6 weeks of gestation who fulfilled the inclusion criteria were recruited. Among the 232 women, 28 patients had positive AF cultures, 82 had an elevated AF IL-6 levels (≥ 1.0 ng/mL), 34 had elevated AF WBC counts (≥ 50 cells/mm^3^), and 56 had histologic chorioamnionitis, and several patients simultaneously met the criteria used in this study for defining IUI (see Supplementary information Table S1 and Fig. 1). Ninety-three women presenting at least one of the IUI criteria were subsequently excluded from the study, leaving a total of 139 women suitable for evaluating the relationship between AF biomarkers and SPTD in the absence of IUI. The characteristics of patients excluded from the study are compared to the analyzed cohort in Supplementary information Table S2. Patients excluded from the study (i.e., women with IUI) delivered significantly earlier and had a higher rate of SPTD at < 34 weeks and ≤ 14 days after sampling than those in the analyzed cohort (i.e., women without IUI).

### Demographic and clinical characteristics of the exploratory cohorts

Characteristics of exploratory cohorts are presented in Table 1. Maternal and gestational ages at sampling were similar in both groups. AF IL-6 levels were higher in the SPTD cases, and a greater proportion of TD controls were nulliparous.

**Table 1:** Demographic and clinical characteristics of women in the exploratory cohorts.

Characteristics	Women delivering at < 34 weeks (n = 10)	Women delivering at term (n = 10)	*P*-value
Age (years)	32.5 (29–41)	31.5 (24–36)	0.280
Nulliparity	40% (4)	100% (10)	0.011
Gestational age at amniocentesis (weeks)	29.8 (26.1–32.5)	29.7 (26.3–32.5)	1.000
AF IL-6 levels (ng/mL)	0.722 (0.243–0.998)	0.112 (0.078–0.236)	< 0.001
AF WBC counts (cells/mm^3^)	2.0 (0–9)	1.50 (0–8)	0.684
Positive AF cultures	0% (0)	0% (0)	
Histologic chorioamnionitis	0% (0)	0% (0)	
Use of tocolytics	100% (10)	100% (10)	
Use of antibiotics	0% (0)	10% (1)	1.000
Use of corticosteroids	100% (10)	60% (6)	0.087
Gestational age at delivery (weeks)	31.5 (29.2–33.1)	38.8 (37.0–40.5)	< 0.001
Cesarean delivery	70% (7)	30% (3)	0.179

Values are given as median (range) or % (n). AF, amniotic fluid; IL, interleukin; WBC, white blood cell.

### Experimental strategy for the discovery and verification of biomarker candidates

**Figure 2:**
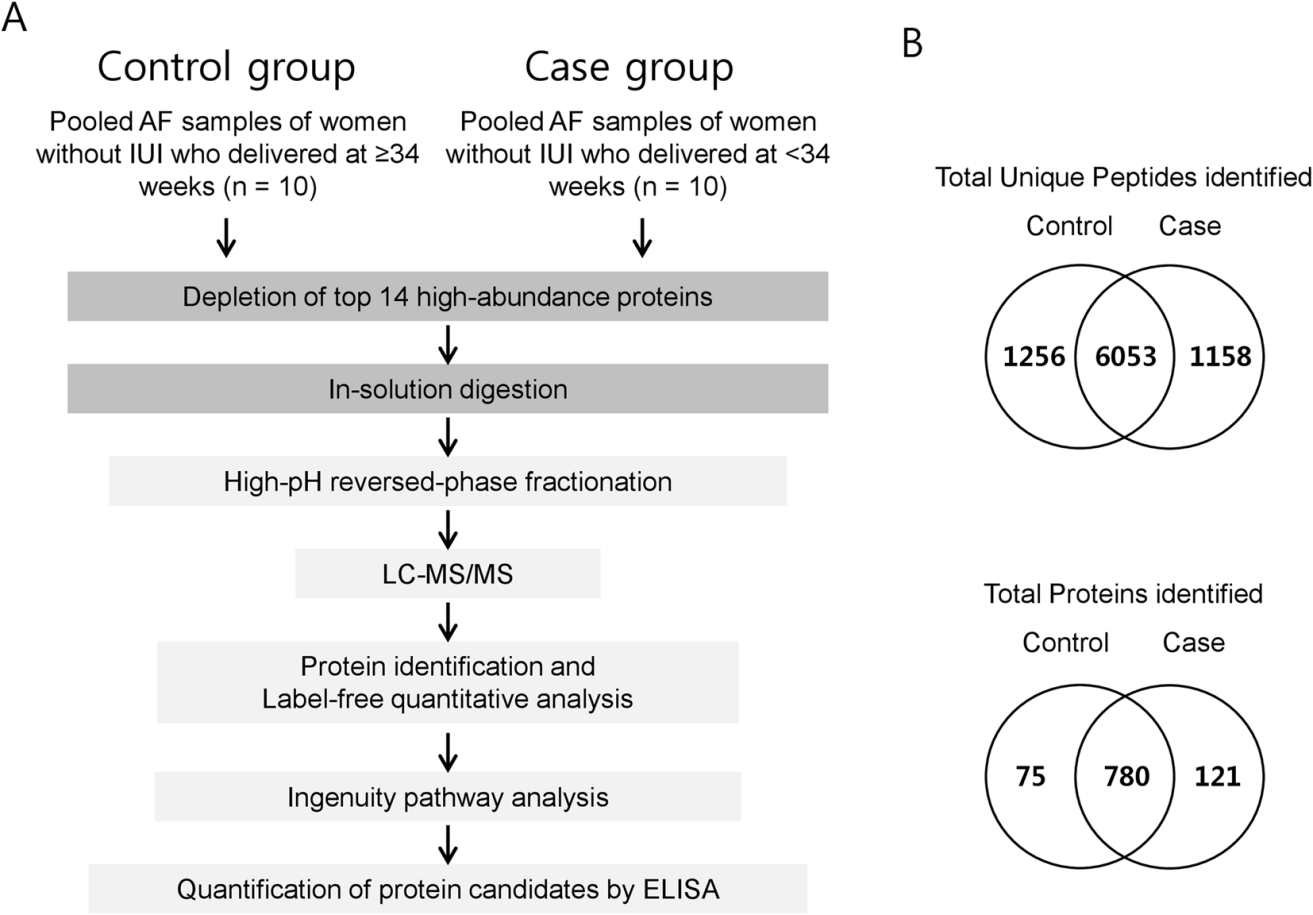
(**A**) Schematic workflow of the experimental design. Amniotic fluid (AF) samples pooled from each group (10 samples per group**)** were subjected to immunoaffinity depletion to remove the 14 most abundant proteins. After high-pH reversed-phase HPLC fractionation of the peptides obtained in the tryptic digestion of each sample group, the peptides were subjected to LC–MS/MS followed by label-free quantitative analysis based on spectral counting. Differentially expressed proteins (DEPs) were functionally annotated using IPA software and the DEPs of interest were validated with ELISA. (**B**) Venn diagrams showing the distribution of unique peptides (above) and proteins (below) identified in the LC–MS/MS analyses. Case: patients without infection/inflammation who delivered at < 34 weeks; Control: patients without infection/inflammation who delivered at term. IUI, intra-uterine infection/inflammation.

Figure 2A outlines the general workflow for identifying AF biomarkers related to SPTD in the absence of infection/inflammation. In the LC-ESI-MS/MS analyses, 7309 and 7211 unique peptides were identified in AF from the control and case groups, respectively, using a 1% FDR threshold at the peptide level, while 6053 peptides were commonly identified in both groups (Fig. 2B). AF from the case and control groups contained 780 common proteins, whereas 855 unique proteins were identified in the control group AF and 901 in the case group (Fig. 2B).

We then applied the following criteria to select differentially expressed proteins (DEPs): (i) *P* value < 0.05 by PLGEM; (ii) log_2_ FC > 0.3; and (iii) spectral counts > 50 from at least one technical replicate of LC-MS/MS run. Seventy-seven DEPs between the SPTD and TD groups met these selection criteria; 48 (62.3%) were upregulated and 29 (37.6%) were downregulated in the SPTD case group when compared to the control group (Supplementary information Table S3). These proteins were further analyzed using IPA (QIAGEN, Redwood City, CA) to determine the associated signaling pathways. As seen in Supplementary information Table S4, the top five canonical pathways were ‘glycolysis I’, ‘gluconeogenesis I’, ‘iron homeostasis signaling pathway’, ‘endoplasmic reticulum stress pathway’, and ‘insulin-like growth factor 1 (IFG-1) signaling’. According to the networks generated from 77 proteins using IPA, the top network (with 32 proteins) contained NF-κB and P38 MAPK as the major hubs (See Supplementary information Figure S1).

### ELISA-based validation of proteomic data in the total cohort based on primary endpoint: SPTD at < 34 weeks

To validate the proteomic analysis findings, quantitative ELISA was conducted to measure AF levels of seven candidate proteins in 139 individual samples. These seven target proteins (CA-1, FCGBP, IGFBP-4, lipocalin-2, S100A8, S100A8/A9, and VEGFR-1) were selected on the basis of potential clinical importance in terms of biological mechanisms reflected by them, statistical significance, and not yet much information on their differential expression associated with SPTD without IUI. The median AF levels of IGFBP-4 and lipocalin-2 were significantly higher and those of VEGFR-1 were significantly lower in women who had SPTD at < 34 weeks than in those who delivered at ≥ 34 weeks (*P* < 0.05 for each, Table 2). Based on this univariate analysis, the high AF FCGBP level had a borderline association with SPTD at < 34 weeks (*P* = 0.079). However, univariate analysis also showed significant correlation between nulliparity or high AF IL-6 levels and SPTD at < 34 weeks (Table 2), so we adjusted the multivariate analyses for baseline differences in nulliparity and AF IL-6 levels. Elevated AF levels of FCGBP and lipocalin-2, as well as lower AF levels of VEFGR-1 were associated with the occurrence of SPTD at < 34 weeks after adjustment for these two variables (Table 3).

**Table 2:** Demographic and clinical characteristics and eight candidate proteins of interest in women from the total cohort.

	SPTD at < 34 weeks (n = 13)	SPTD at ≥ 34 weeks (n = 126)	*P*-value
Age (years)	32.62 ± 4.90	31.18 ± 3.65	0.573
Nulliparity	7 (53.8%)	95 (75.4%)	0.025
Gestational age at amniocentesis (weeks)	29.39 ± 2.22	29.78 ± 2.38	0.434
AF IL-6 levels (ng/mL)	0.547 ± 0.268	0.306 ± 0.235	0.001
AF WBC counts (cells/mm^3^)	2.92 ± 3.43	4.21 ± 5.14	0.358
Positive AF cultures	0 (0%)	0 (0%)	1.000
Use of tocolytics	12 (92.3%)	115 (91.3%)	0.899
Use of corticosteroids	12 (92.3%)	72 (57.1%)	0.014
Use of antibiotics	0 (0%)	26 (20.6%)	0.070
Gestational age at delivery (weeks)	30.30 ± 2.22	37.93 ± 1.87	< 0.001
Cesarean delivery^a^	9/13 (69.2%)	35/118 (29.7%)	0.010
AF CA-1 (ng/mL)	2.82 ± 2.84	1.66 ± 1.60	0.364
AF FCGBP (ng/mL)	48.55 ± 19.83	37.94 ± 16.87	0.079
AF IGFBP-4 (ng/mL)	664.12 ± 254.09	525.27 ± 236.22	0.033
AF lipocalin-2 (ng/mL)	402.65 ± 484.37	193.42 ± 219.57	0.002
AF S100 A8 (ng/mL)	1.64 ± 2.01	1.06 ± 0.93	0.309
AF S100 A8/A9 (ng/mL)	2465.36 ± 741.84	2321.12 ± 792.29	0.466
AF VEGFR-1 (ng/mL)	496.75 ± 423.96	1062.02 ± 693.29	< 0.001

Values are given as the mean ± standard deviation or n (%). SPTD, spontaneous preterm delivery; AF, amniotic fluid; WBC, white blood cells; CA-1, carbonic anhydrase 1; FCGBP Fc fragment of IgG binding protein; IGFBP, insulin-like growth factor-binding protein; S100A8, S100 calcium binding protein A8; VEGFR-1, vascular endothelial growth factor receptor 1. ^a^ Eight cases were excluded for the analysis because delivery took place at term at another institution and the mode of delivery was unknown.

**Table 3:** Relationship between various proteins in the amniotic fluid and spontaneous preterm delivery at < 34 weeks or ≤ 14 days of sampling in women with preterm labor without infection and/or inflammation, analyzed by multiple logistic regressions.

Variables	Spontaneous preterm delivery < 34 weeks		Spontaneous preterm delivery within 14 days	
	Adjusted ^a^	*P*-value ^b^	Adjusted ^c^	*P*-value ^d^
AF FCGBP (ng/mL)	1.041 (1.001–1.082)	0.046	1.048 (1.006–1.091)	0.025
AF IGFBP-4 (ng/mL)	1.002 (1.000–1.005)	0.065		
AF lipocalin-2 (ng/mL)	1.002 (1.000–1.003)	0.035	1.001 (0.999–1.002)	0.549
AF VEGFR-1 (ng/mL)	0.998 (0.996–1.000)	0.042	0.997 (0.995–1.000)	0.029

AF, amniotic fluid; FCGBP, Fc fragment of IgG binding protein; IGFBP, insulin-like growth factor-binding protein; VEGFR-1, vascular endothelial growth factor receptor 1.

^a^Adjustment for nulliparity and AF interleukin-6 levels. ^b^ For the adjusted odds ratio. ^c^ Adjustment for AF interleukin-6 levels. ^d^ For the adjusted odds ratio.

### ELISA-based validation of proteomic data using ELISA in the total cohort based on secondary endpoint: SPTD within 14 days of sampling

Similarly to the primary endpoint results, the median AF concentrations of FCGBP and lipocalin-2 were significantly higher and those of VEGFR-1 were significantly lower in women who experienced SPTD within 14 days of sampling than in those who delivered after these 14 days (*P* < 0.05 for each, Table 4). However, although all women analyzed in this study had AF IL-6 levels of less than 1.0 ng/mL, women who had SPTD within 14 days of sampling had a significantly higher AF IL-6 level than those who delivered after this period. Accordingly, we adjusted the multivariate analyses for AF IL-6 levels and found that elevated FCGBP levels and lower VEFGR-1 levels in AF were significantly associated with the risk of SPTD ≤ 14 days after sampling, independently of the AF IL-6 level (Table 3).

**Table 4:** Demographic and clinical characteristics and eight candidate proteins of interest in women from the total cohort (n = 139). SPTD, spontaneous preterm delivery; AF, amniotic fluid; WBC, white blood cells; CA-1, carbonic anhydrase 1; FCGBP Fc fragment of IgG binding protein; IGFBP, insulin-like growth factor-binding protein; S100A8, S100 calcium binding protein A8; VEGFR-1, vascular endothelial growth factor receptor 1. Values are given as the mean ± standard deviation or n (%). ^a^Eight cases were excluded for the analysis because delivery took place at term at another institution and the mode of delivery was unknown**.**

	SPTD ≤ 14 days (n = 10)	SPTD > 14 days (n = 129)	*P*-value
Age (years)	31.20 ± 4.16	31.33 ± 3.78	0.561
Nulliparity	6 (60.0%)	95 (73.6%)	0.353
Gestational age at amniocentesis (weeks)	30.08 ± 2.40	29.71 ± 2.36	0.590
AF IL-6 levels (ng/mL)	0.565 ± 0.276	0.310 ± 0.237	0.003
AF WBC counts (cells/mm^3^)	2.90 ± 3.54	4.18 ± 5.10	0.393
Positive AF cultures	0 (0%)	0 (0%)	1.000
Use of tocolytics	9 (90.0%)	118 (91.5%)	0.874
Use of corticosteroids	9 (90.0%)	75 (58.1%)	0.048
Use of antibiotics	1 (10.0%)	25 (19.4%)	0.465
Gestational age at delivery (weeks)	30.47 ± 2.71	37.75 ± 2.21	< 0.001
Cesarean delivery ^a^	5/10 (50.0%)	39/121 (32.2%)	0.302
AF CA-1 (ng/mL)	2.25 ± 2.20	1.73 ± 1.74	0.954
AF FCGBP (ng/mL)	51.96 ± 16.70	37.84 ± 17.02	0.018
AF IGFBP-4 (ng/mL)	628.86 ± 294.29	531.24 ± 235.67	0.304
AF lipocalin-2 (ng/mL)	259.79 ± 152.89	209.36 ± 266.98	0.040
AF S100 A8 (ng/mL)	1.18 ± 0.80	1.11 ± 1.10	0.625
AF S100 A8/A9 (ng/mL)	2466.91 ± 734.50	2324.36 ± 791.91	0.551
AF VEGFR-1 (ng/mL)	464.50 ± 256.43	1051.37 ± 697.18	0.002

## Discussion

In this study of women with PTL in the absence of IUI, we (1) characterized 77 DEPs in the AF of women who spontaneously delivered preterm (< 34 weeks) using a label-free LC-MS/MS analysis; (2) found that five principle pathways associated with the 77 DEPs were glycolysis, gluconeogenesis, iron homeostasis signaling, the endoplasmic reticulum stress pathway, and IFG-1 signaling; and (3) validated selected targets (7 DEPs) with significant up or downregulation in the AF of women with SPTD at < 34 weeks compared to women with SPTD at ≥ 34 weeks. FCGBP, lipocalin-2, and VEGFR-1 levels in AF were independently associated with SPTD at < 34 weeks in the absence of IUI.

The data presented here in the context of SPTD without IUI reveals plausible mechanisms of idiopathic preterm labor and delivery. Notwithstanding the exclusion of IUI cases from the study, an inflammatory response was still present as a disease or disorder associated to SPTD (Supplementary information Table S4). Similarly, previous research showed strong evidence that the inflammatory pathway may be a part of the primary stimuli that initiate the preterm labor cascade in the absence of infection/inflammation, such as in cases of uterine overdistension, maternal stress, and the decline in progesterone action^3,22–24^. Moreover, inflammatory pathways have also been considered to be implicated in the initiation of normal term labor^3,25^. Taken together, our and other groups’ findings suggest that inflammation may be a common pathway that leads to PTL and SPTD, regardless of the several distinct clinical phenotypes of preterm birth syndrome^3,22–24^. Our findings that patients with IUI delivered significantly earlier and had higher rates of SPTD at < 34 weeks and ≤ 14 days after sampling than those without IUI (Table S2) are consistent with the observations of Catov et al. and Manuck et al., who demonstrated that preterm births with inflammation/infection happened earlier than preterm births with no lesions ^26,27^. We chose SPTD before 34 weeks and SPTD within 14 days of sampling as the primary and secondary endpoints, respectively. The former outcome is closely related to the incidence of neonatal morbidity and mortality in relation to prematurity^28^. The latter outcome is associated with the need for hospitalization, tertiary care center referral, and treatments (e.g., administration of corticosteroids, neuroprotection).

In the current study, an important observation that merits attention is that VEGFR-1, FCGBP, and lipocalin-2 in AF are potential independent novel biomarkers for non-infectious and non-inflammatory SPTD. VEGFR-1 is expressed in the placenta and amnion, and is produced by extravillous and villous trophoblasts^29^. The soluble form of VEGFR-1 (sVEGFR-1) has strong anti-angiogenic activity through its ligand-binding to all isoforms of VEGF and placental growth factor^29^. Previous research has shown that sVEFGR-1 levels in serum and AF of women with preeclampsia are elevated, and that sVEFGR-1 is specifically responsible for inhibiting placental angiogenesis^30,31^. In the context of PTL, a recent study has demonstrated that decreased AF levels of sVEGFR-1 in the second trimester are associated with subsequent SPTD^32^, which is consistent with our results. Data from our and other groups^32^ suggest that altered angiogenic responses in the amniotic cavity may have an important biological role in the pathophysiology of SPTD. FCGBP is a large protein (over 500 kDa) located in the mucosa of endodermal-derived tissues (e.g., cervix and intestine) that plays a major role in mucosal immunological defenses^33,34^. In particular, FCGBP has been identified as altered in several cancers^35,36^, suggesting its important role in the maintenance of homeostasis. However, to date, no studies have examined whether the changes in AF FCGBP levels are associated with SPTD. In the present study, we have shown for the first time that an elevated FCGBP level in AF was associated with SPTD before 34 weeks and within 14 days of sampling in women with PTL and no IUI.

Lipocalin-2, known as neutrophil gelatinase-associated lipocalin, is mainly produced in the amniochorion during pregnancy and is involved in the modulation of both innate and adaptive immune responses, as well as in the induction of apoptosis in hematopoietic cells^37^. In particular, lipocalin-2 has been recently characterized as an adipose-derived cytokine, contributing to a low-grade inflammatory state in obese individuals^37^. In line with the known biology and function of lipocalin-2, AF levels of this protein have been reported as increased in pregnancies complicated by MIAC/histologic chorioamnioitis, IAI and preterm birth^38,39^. These results are in agreement with those of the present study. The same results obtained in studies conducted with patients with or without infection/inflammation suggested that the main pathways associated with SPTD are not clearly divided into different clusters and may have a common pathway, perhaps related to the inflammatory response.

IGFBP-4 is a member of a family of IGFBPs that regulates IGF activity, which is involved in the control mechanism of placental and fetal growth and development and is highly expressed in placental syncytiotrophoblasts^40,41^. Maternal serum levels of IGFBP-4 in early pregnancy have been reported to be significantly altered in cases with later development of fetal growth restriction (FGR) when compared to normal pregnancies, thus confirming its function as an enhancer of placental growth and development^42^. In a recent study on maternal serum, Saade et al. have demonstrated that an elevated serum IGFBP-4 level at 17–28 weeks may be a useful predictor for identifying asymptomatic pregnant women at risk of SPTD^43^, similarly to the results obtained in the current study using AF samples. Thus, we speculated that an elevated level of IGFBP-4 in women with SPTD may reflect placental dysfunction, particularly owing to maternal vascular insult, and thus may be related to an increased risk of SPTD.

In this study, IPA analyses allowed the identification of 5 pathways potentially involved in PTL and SPTD in the absence of IUI: glycolysis, gluconeogenesis, endoplasmic reticulum stress pathway, iron homeostasis and IGF-1 signaling. These are in accordance with previous proteomic studies that used plasma taken between 10 and 15 weeks’ gestation or AF samples^6,44^. In this study, we have shown an important role for glucose metabolism (glycolysis and gluconeogenesis) in PTL and SPTD without IUI, similarly to a report that demonstrated the role of diabetes pathways in the development of SPTD later in pregnancy^44^. In fact, these findings are not unexpected because preterm parturition has been linked to the many cellular processes that use and produce energy, and consequently glucose utilization and production were significantly increased throughout parturition^45^. Naturally, associations between low AF glucose levels and MIAC or preterm delivery have been reported^46,47^. Differential expression of AF proteins involved in iron homeostasis (iron homeostasis signaling pathway) is expected in cases in which PTL and SPTD are owing to placental abruption or decidual hemorrhage, which may result in high levels of free iron and heme in the AF. An endoplasmic reticulum stress pathway known as the unfolded protein response (UPR) is important for normal cellular homeostasis and for organism development^48^. Endoplasmic reticulum stress and evidence of UPR are observed in placenta from preeclampsia and FGR pregnancies and in the inflammatory responses associated with labor or infection in fetal membranes and myometrium of pregnant women with term and preterm deliveries^49,50^. Therefore, in light of the mechanisms leading to PTL and SPTD without IUI, endoplasmic reticulum stress pathways may be explained as reflecting the placental vascular pathology that leads to preterm parturition. Similarly, alterations in the expression of proteins involved in the IGF-1 signaling pathway could be ascribed to SPTD due to abnormal placentation with insufficient trophoblastic invasion, as aforementioned^40–42^. Although in the present study five principle pathways related to DEPs were specifically revealed based on IPA analysis, further ex vivo studies are needed to clarify the precise mechanistic role of these proteins in pathways related to SPTD in the absence of IUI.

This study has several limitations to be considered. First, although we tried to select only women without IUI using the criteria based on both AF analyses and placental pathology, there is still a statistical difference in AF IL-6 levels between SPTL and TD groups, suggesting that patients with possible IUI may not have been completely excluded from the analyzed cohort. Second, the present study is of a retrospective nature, was performed at a single center, and ELISA validation was not conducted on a completely independent cohort due to the limited number of SPTD samples. All of these reasons may decrease the generalizability of our results to other cohorts. Third, we did not use polymerase chain reaction (PCR) to exclude microbial invasion of the amniotic cavity (MIAC), despite the fact that PCR and culture-based methods are complementary techniques in MIAC detection^51^. However, it is unlikely that this has changed our main findings because besides AF culture, three other exclusion criteria (AF IL levels, AF WBC counts, and histologic chorioamnionitis) were applied to more appropriately select PTL cases caused by non-IUI reasons. In fact, positive PCR/ESI–MS (≥ 17 GE/well) and negative AF cultures are reportedly associated with increased rates of intra-amniotic inflammation (AF IL-6 levels ≥ 2.6 ng/mL) and acute histologic chorioamnionitis, thus occurring in 90% (9/10) and 70% (7/10) of the PTL patients with this condition**,** respectively^52^. Fourth, it is possible that the proteins identified as DEPs in this proteomic study may not be reproducible since only 4 of the 7 (57%) proteins found by proteomics were confirmed by ELISA in individual samples from the total cohort. However, in this regard, it can be explained that the control group in proteomic experiments contained 10 women with TD (≥ 37 weeks), whereas control group for ELISA was comprised of 126 women who delivered at ≥ 34 weeks. Fifth, only a small number of the STPD in the absence of IUI were available in the present study, which prevented a definitive conclusion from these data. Sixth, our study used frozen stored samples to measure AF IL-6 levels. This could lead to alterations in the AF IL-6 levels. Thus, these AF IL-6 levels may be different from those found at the time of an episode of PTL, as suggested by a previous report^53^. Seventh, we used a single proinflammatory cytokine (i.e., IL-6), rather than multiple proinflammatory cytokines, to exclude PTL patients with possible IUI, despite a recent study that has reported that PTL patients can have a network of different inflammatory-related proteins in the AF according to the presence or absence of MIAC and/or intra-amniotic inflammation. This may prevent complete exclusion of possible IUI^54^. The main strength of the study is that PTL cases caused by non-IUI reasons were more strictly selected for the present study in comparison to other similar studies^6–9^, because we have selected cases based on information on both AF analyses and placental pathology findings.

## Conclusions

Using a proteomic analysis of AFs from women with PTL, we have discovered several potential novel biomarkers (i.e., FCGBP, lipocalin-2, and VEGFR-1) and specific signaling pathways related to SPTD in the absence of IUI. These altered proteins in AF may offer potential therapeutic targets to prevent SPTD.

## Supplementary information

Supplementary Tables.

Supplementary Information.

Supplementary Legend.

## Data Availability

All relevant data are within the paper, and the authors can make available materials, data and associated protocols if requested.
