# Supplementary material for: Identifying potential biomarkers related to pre-term delivery by proteomic analysis of amniotic fluid
Source: Sci Rep. 2020 Nov 12;10:19648. doi: 10.1038/s41598-020-76748-1 (PMC7665029; doi:10.1038/s41598-020-76748-1)
Supplement: Supplementary file 1 — Supplementary Tables. [file 41598_2020_76748_MOESM1_ESM.doc]

**Title page**

**Amniotic fluid proteomic analysis to identify potential biomarkers for preterm birth in women with preterm labor without cultivable** bacterial **infection/inflammation**

Subeen Hong^1,†^, Ji Eun Lee^2,†^, Yu Mi Kim^3^, Yehyon Park^3^, Ji-Woong Choi^4^, and Kyo Hoon Park^3^,**^*^**

^1^ Department of Obstetrics and Gynecology, College of Medicine, The Catholic University of Korea, Seoul, Korea

^2^ Center for Theragnosis, Biomedical Research Institute, Korea Institute of Science and Technology, Seoul, Korea

^3^ Department of Obstetrics and Gynecology, Seoul National University College of Medicine, Seoul National University Bundang Hospital, Seongnam, Korea

^4^ Wide River Institute of Immunology, Seoul National University, Hongcheon, Korea

^†^ These two authors contributed equally to this work and should therefore be regarded as equivalent authors.

**^*^** Corresponding author

Address correspondence to:

Kyo Hoon Park, MD, PhD

Department of Obstetrics and Gynecology

Seoul National University Bundang Hospital

82, Gumi-ro 173 Beon-gil, Bundang-gu, Seongnam, 463-707, KoreaTel: 82-31-787-7252; Fax: 82-31-787-4054; E-mail: [pkh0419@snubh.org](mailto:pkh0419@snubh.org)

| Characteristics | Number of cases |
| --- | --- |
| AF interleukin-6 ≥ 1.0 ng/mL | 82 (88.1%) |
| AF white blood cell ≥ 50 cells/mm^3^ | 34 (36.5%) |
| Positive AF culture | 28 (30.1%) |
| Histologic chorioamnionitis^a^ | 56 (65.1%, 56/86) |

**Table S1.** Reasons for the exclusion of women based on the diagnostic criteria for infection/inflammation (n = 93). AF, amniotic fluid. Values are given as n (%). ^a^Data for the histologic evaluation of the placenta were only available in 86 of the 93 women because histologic evaluation of the placenta was not performed in 7cases because of our institutional policy that only the placentas in cases of preterm delivery are to be sent for histopathologic examination.

|  | Women with infection/inflammation (n = 93) | Women without infection/inflammation  (n = 139) | *P*-value |
| --- | --- | --- | --- |
| Age (years) | 31.38 ± 3.93 | 31.32 ± 3.79 | 0.902 |
| Nulliparity | 50 (53.8%) | 101 (72.7%) | 0.003 |
| Gestational age at amniocentesis (weeks) | 29.74 ± 2.35 | 29.23 ± 2.25 | 0.099 |
| Amniotic fluid IL-6 levels (ng/mL) | 16.961 ± 25.274 | 0.335 ± 0.244 | <0.001 |
| Amniotic fluid WBC counts (cells/mm^3^) | 1395.61 ± 3794.49 | 4.09 ± 5.01 | 0.001 |
| Positive amniotic fluid cultures | 28 (30.1%) | 0 (0%) | <0.001 |
| Histologic chorioamnionitis^a^ | 57/86 (66.3%) | 0/36 (0%) | <0.001 |
| Use of tocolytics | 91 (97.8%) | 127 (91.4%) | 0.042 |
| Use of corticosteroids | 81 (87.1%) | 84 (60.4%) | <0.001 |
| Use of antibiotics | 45 (48.4%) | 26 (18.7%) | <0.001 |
| Gestational age at delivery (weeks) | 31.74 ± 3.90 | 37.22 ± 2.93 | <0.001 |
| SPTD ≤14 days | 61 (65.6%) | 10 (7.2%) | <0.001 |
| SPTD at <34 weeks | 66 (71.0%) | 13 (9.4%) | <0.001 |
| Cesarean delivery ^a^ | 30/93 (32.3%) | 44/131 (33.6%) | 0.835 |

**Table S2.** Comparison between characteristics and delivery outcomes of the analyzed cohort (i.e., women without infection/inflammation) and those of patients excluded from the study (i.e., women with infection/inflammation). IL, interleukin; WBC, white blood cell; SPTD, spontaneous preterm delivery. Values are given as the mean ± standard deviation or n (%). ^a^Data for the histologic evaluation of the placenta were only available in 139 of the 232 women because in 8 cases, delivery took place at another institution and in 102 cases, histologic evaluation of the placenta was not performed because of our institutional policy that only the placentas in cases of preterm delivery are to be sent for histopathologic examination. ^a^ Eight cases were excluded for the analysis because delivery took place at term at another institution and the mode of delivery was unknown.

| Accession number | | Protein description | Gene name | Log2FC ratio | *P*-value |
| --- | --- | --- | --- | --- | --- |
| P68871 | Hemoglobin subunit beta | | HBB | 1.84 | 0.00009 |
| P13796 | Plastin-2 | | LCP1 | 1.06 | 0.0001 |
| P13727 | Bone marrow proteoglycan | | PRG2 | -0.52 | 0.00035 |
| P08833 | Insulin-like growth factor-binding protein 1 | | IGFBP1 | 0.86 | 0.00046 |
| D6RE86 | Ceruloplasmin (Fragment) | | CP | -0.68 | 0.00055 |
| B4DWR5 | cDNA FLJ51358, highly similar to Involucrin | | N/A | -1.31 | 0.00057 |
| P51884 | Lumican | | LUM | 0.34 | 0.00087 |
| A0A087WW43 | Inter-alpha-trypsin inhibitor heavy chain H3 | | ITIH3 | 0.58 | 0.00174 |
| P22692 | Insulin-like growth factor-binding protein 4 | | IGFBP4 | 0.57 | 0.00187 |
| P06733 | Alpha-enolase | | ENO1 | 0.47 | 0.00189 |
| Q06033-2 | Isoform 2 of Inter-alpha-trypsin inhibitor heavy chain H3 | | ITIH3 | 0.55 | 0.0019 |
| P15924 | Desmoplakin | | DSP | 0.47 | 0.00193 |
| P07476 | Involucrin | | IVL | -0.58 | 0.00211 |
| P13727-2 | Isoform 2 of Bone marrow proteoglycan | | PRG2 | -0.55 | 0.00227 |
| P18669 | Phosphoglycerate mutase 1 | | PGAM1 | 0.84 | 0.00228 |
| B4DN75 | cDNA FLJ60724, highly similar to Cartilage oligomeric matrix protein | | N/A | 0.42 | 0.00247 |
| P35858-2 | Isoform 2 of Insulin-like growth factor-binding protein complex acid labile subunit | | IGFALS | -0.42 | 0.00247 |
| P26038 | Moesin | | MSN | 1 | 0.0025 |
| P69892 | Hemoglobin subunit gamma-2 | | HBG2 | 0.86 | 0.00297 |
| O00391 | Sulfhydryl oxidase 1 | | QSOX1 | -0.32 | 0.00335 |
| P29622 | Kallistatin | | SERPINA4 | -0.45 | 0.00411 |
| Q96PD5 | N-acetylmuramoyl-L-alanine amidase | | PGLYRP2 | -0.71 | 0.00423 |
| Q02487-2 | Isoform 2B of Desmocollin-2 | | DSC2 | 0.35 | 0.00452 |
| P04745 | Alpha-amylase 1 | | AMY1C | 0.92 | 0.00462 |
| P00558 | Phosphoglycerate kinase 1 | | PGK1 | 1.46 | 0.00547 |
| Q6S8J3 | POTE ankyrin domain family member E | | POTEE | 0.73 | 0.00571 |
| P11465 | Pregnancy-specific beta-1-glycoprotein 2 | | PSG2 | -0.35 | 0.00596 |
| P32926 | Desmoglein-3 | | DSG3 | 0.68 | 0.00646 |
| P01034 | Cystatin-C | | CST3 | -0.83 | 0.00669 |
| A0A087WXI2 | IgGFc-binding protein | | FCGBP | 4.32 | 0.0072 |
| Q53FR6 | Cartilage oligomeric matrix protein variant (Fragment) | | N/A | 0.44 | 0.00808 |
| B4DWU6 | cDNA FLJ51361, highly similar to Keratin, type II cytoskeletal 6A | | N/A | 0.58 | 0.00836 |
| Q00887 | Pregnancy-specific beta-1-glycoprotein 9 | | PSG9 | -0.91 | 0.00844 |
| P07358 | Complement component C8 beta chain | | C8B | -0.48 | 0.0095 |
| Q6P988 | Palmitoleoyl-protein carboxylesterase NOTUM | | NOTUM | -0.4 | 0.01164 |
| P05109 | Protein S100-A8 | | S100A8 | -0.4 | 0.01246 |
| Q5SZK8 | FRAS1-related extracellular matrix protein 2 | | FREM2 | 1.02 | 0.01287 |
| P25311 | Zinc-alpha-2-glycoprotein | | AZGP1 | -0.39 | 0.01335 |
| P62805 | Histone H4 | | HIST4H4 | 1.55 | 0.0135 |
| B2R7Y0 | cDNA, FLJ93654, highly similar to Homo sapiens serpin peptidase inhibitor, clade B (ovalbumin), member 2 (SERPINB2), mRNA | | N/A | 0.44 | 0.01373 |
| P19801-2 | Isoform 2 of Amiloride-sensitive amine oxidase [copper-containing] | | AOC1 | -0.42 | 0.01539 |
| P02788-2 | Isoform DeltaLf of Lactotransferrin | | LTF | 0.38 | 0.0154 |
| P80188 | Neutrophil gelatinase-associated lipocalin | | LCN2 | 0.91 | 0.01568 |
| P60174-1 | Isoform 2 of Triosephosphate isomerase | | TPI1 | 0.68 | 0.01577 |
| P11021 | 78 kDa glucose-regulated protein | | HSPA5 | 0.61 | 0.01599 |
| Q9BXP8 | Pappalysin-2 | | PAPPA2 | -0.35 | 0.01704 |
| P10451-3 | Isoform C of Osteopontin | | SPP1 | 0.39 | 0.01799 |
| P05120 | Plasminogen activator inhibitor 2 | | SERPINB2 | 0.41 | 0.02117 |
| P26927 | Hepatocyte growth factor-like protein | | MST1 | -0.58 | 0.02143 |
| O95633-2 | Isoform 2 of Follistatin-related protein 3 | | FSTL3 | -0.47 | 0.0217 |
| P69905 | Hemoglobin subunit alpha | | HBA2 | 0.99 | 0.0222 |
| P04196 | Histidine-rich glycoprotein | | HRG | -0.49 | 0.0222 |
| P00915 | Carbonic anhydrase 1 | | CA1 | 2.71 | 0.02262 |
| P02042 | Hemoglobin subunit delta | | HBD | 2.39 | 0.0231 |
| P10451-5 | Isoform 5 of Osteopontin | | SPP1 | 0.34 | 0.02325 |
| P07737 | Profilin-1 | | PFN1 | 1.4 | 0.02472 |
| Q53FV4 | Lumican variant (Fragment) | | N/A | 1.43 | 0.02473 |
| P15151-2 | Isoform Beta of Poliovirus receptor | | PVR | 0.54 | 0.02535 |
| P10451-2 | Isoform B of Osteopontin | | SPP1 | 0.32 | 0.0274 |
| P15311 | Ezrin | | EZR | 0.4 | 0.02751 |
| O00468-6 | Isoform 6 of Agrin | | AGRN | 0.56 | 0.02757 |
| Q13219 | Pappalysin-1 | | PAPPA | -0.46 | 0.02777 |
| P13797 | Plastin-3 | | PLS3 | 0.76 | 0.02831 |
| P09486 | SPARC | | SPARC | 0.38 | 0.03224 |
| P17948-4 | Isoform 4 of Vascular endothelial growth factor receptor 1 | | FLT1 | -0.37 | 0.03228 |
| P17948 | Vascular endothelial growth factor receptor 1 | | FLT1 | -0.3 | 0.03241 |
| Q99715 | Collagen alpha-1(XII) chain | | COL12A1 | -0.37 | 0.03275 |
| H3BUX1 | Mesothelin (Fragment) | | MSLN | 0.33 | 0.03368 |
| Q59FF1 | Insulin-like growth factor binding protein 2 variant (Fragment) | | N/A | 0.63 | 0.03533 |
| P13646 | Keratin, type I cytoskeletal 13 | | KRT13 | 0.38 | 0.03555 |
| A0A087WYD8 | Amine oxidase | | AOC1 | -0.37 | 0.03624 |
| P0DML2 | Chorionic somatomammotropin hormone 1 | | CSH1 | -0.36 | 0.04079 |
| P20061 | Transcobalamin-1 | | TCN1 | -0.49 | 0.04131 |
| P27797 | Calreticulin | | CALR | 0.36 | 0.04229 |
| G9K389 | YWHAE/FAM22B fusion protein (Fragment) | | YWHAE/FAM22B fusion | 0.36 | 0.04526 |
| B4DWS3 | cDNA FLJ60407, highly similar to Serine protease inhibitor Kazal-type 5 (Fragment) | | N/A | 0.42 | 0.04704 |
| B2R8I2 | cDNA, FLJ93914, highly similar to Homo sapiens histidine-rich glycoprotein (HRG), mRNA | | N/A | -0.43 | 0.04835 |

**Table S3.** List of 77 amniotic fluid proteins that exhibited significant changes in pairwise comparison between spontaneous preterm labor and delivery (< 34 weeks) and term delivery in women without infection and/or inflammation.

| **Diseases and disorders** | *P*-value^†^ | | No.^‡^ |
| --- | --- | --- | --- |
| Dermatological diseases and conditions | 9.20E-03 - 4.92E-10 | | 55 |
| Inflammatory disease | 1.22E-02 - 4.92E-10 | | 29 |
| Inflammatory response | 1.22E-02 - 4.92E-10 | | 35 |
| Organismal injury and abnormalities | 1.22E-02 - 4.92E-10 | | 62 |
| Hereditary disorders | 1.22E-02 - 1.68E-08 | | 28 |
| **Molecular and cellular functions** | *P*-value^†^ | | No.^‡^ |
| Protein synthesis | 4.24E-07 - 3.84E-15 | | 16 |
| Cellular compromise | 6.14E-03 - 7.86E-10 | | 20 |
| Cellular movement | 1.22E-02 - 1.26E-07 | | 24 |
| Carbohydrate metabolism | 9.97E-03 - 3.24E-07 | | 9 |
| Cell morphology | 1.22E-02 - 2.30E-06 | | 16 |
| **Associated network functions** |  | | Score |
| Cellular movement, cell death and survival, cellular assembly and organization |  | | 60 |
| Gene expression, cell death and survival, organismal survival |  | | 25 |
| **Top canonical pathways** | *P*-value^†^ | | Ratio |
| Glycolysis I | 8.22E-07 | | 4/24 (0.167) |
| Gluconeogenesis I | 6.07E-05 | | 3/25 (0.120) |
| Iron homeostasis signaling pathway | 7.09E-04 | | 4/131 (0.031) |
| Endoplasmic reticulum stress pathway | 1.88E-03 | | 2/21 (0.095) |
| IGF -1 signaling | 4.15E-03 | 3/105 (0.029) | |

**Table S4.** Summary of the ingenuity pathway analysis for the 77 proteins with altered expression in spontaneous preterm labor and delivery (< 34 weeks) in comparison to term delivery in women without infection and/or inflammation. ^†^ *P*-values are displayed in E notation: aEb indicates a value of a × 10^b^. ^‡^ Numbers of molecules involved.
