# Supplementary material for: Identifying potential biomarkers related to pre-term delivery by proteomic analysis of amniotic fluid
Source: Sci Rep. 2020 Nov 12;10:19648. doi: 10.1038/s41598-020-76748-1 (PMC7665029; doi:10.1038/s41598-020-76748-1)
Supplement: Supplementary file 2 — Supplementary Information. [file 41598_2020_76748_MOESM2_ESM.pdf]

Title page

Amniotic fluid proteomic analysis to identify potential biomarkers for preterm birth in women with preterm labor without cultivable bacterial infection/inflammation

Subeen Hong[1,†], Ji Eun Lee[2,†], Yu Mi Kim[3], Yehyon Park[3], Ji-Woong Choi[4], and Kyo Hoon Park[3,*]

[1] Department of Obstetrics and Gynecology, College of Medicine, The Catholic University of Korea, Seoul, Korea

[2] Center for Theragnosis, Biomedical Research Institute, Korea Institute of Science and Technology, Seoul, Korea

[3] Department of Obstetrics and Gynecology, Seoul National University College of Medicine, Seoul National University Bundang Hospital, Seongnam, Korea

[4] Wide River Institute of Immunology, Seoul National University, Hongcheon, Korea

[†] These two authors contributed equally to this work and should therefore be regarded as equivalent authors.

[*] Corresponding author

Address correspondence to:
Kyo Hoon Park, MD, PhD
Department of Obstetrics and Gynecology
Seoul National University Bundang Hospital
82, Gumi-ro 173 Beon-gil, Bundang-gu, Seongnam, 463-707, KoreaTel: 82-31-787-7252;

Fax: 82-31-787-4054; E-mail: pkh0419@snubh.org

- Supplemental Materials –

Management of preterm labor

The use and choice of a tocolytic drug, management of intra-amniotic infection/inflammation (IAI), and the decision of when to deliver a baby of a PTL patient were left to the discretion of attending physicians. Women with PTL were treated initially with hydration. If uterine contractions persisted, they received continuous intravenous tocolytic therapy with magnesium sulfate, ritodrine, or atosiban. Magnesium sulfate and ritodrine were the first-line tocotytics in our hospital because the use of atociban is covered by public insurance in Korea only when magnesium sulfate and ritodrine are contraindicated, fail, or cause side effects. At our institution, women with PTL were not given antibiotics to prolong pregnancy, except for development of clinical signs of chorioamnionitis and treatment of clinically diagnosed (or suspected) IAI. Maternal or fetal health status was carefully monitored for the development of clinical signs of chorioamnionitis and/or fetal compromise, both of which are indications for induction of labor. In women with a diagnosis of intra-amniotic infection (i.e., positive AF cultures) but without clinical chorioamnionitis, antibiotics effective against isolated bacteria were administered. Clinical signs of chorioamnionitis and/or fetal compromise were closely monitored until 34 weeks of gestation. Induction of labor was considered after 34 weeks if there was no development of clinical signs of chorioamnionitis, fetal compromise, or labor. Corticosteroids were administered between 24 and 34 weeks of gestation to mature fetal lungs.

Immunoaffinity depletion of high-abundance proteins

The top 14 high-abundance proteins (albumin, alpha-1 acid glycoprotein, alpha-1 antitrypsin, alpha-2 macroglobulin, apolipoprotein A-I, apolipoprotein A-II, complement C3, fibrinogen, haptoglobin, immunoglobulin A, immunoglobulin G, immunoglobulin M, serotransferrin, transthyretin) were removed from 200 μg of each pooled sample using a multiple affinity removal system (MARS) (Hu-14; Agilent Technologies, Santa Clara, CA) according to the manufacturer's instructions. The immuno-depleted sample solutions were exchanged with 50 mM ammonium bicarbonate (ABC), and concentrated with a 3 kDa molecular cut-off filter (Amicon Ultra centrifugal filter units, Ultracel-3K; Millipore, Billerica, MA). The total protein concentration was further determined via BCA assay.

In-solution tryptic digestion

The 75 μg of protein sample from the depleted AF in each group was diluted by 8 M Urea in 50 mM ABC and samples were reduced with 100 mM dithiothreitol and alkylated with 100 mM iodoacetamide for 1 h at room temperature in the dark. Prior to digestion with trypsin (Promega, Madison, WI), the samples were diluted to 0.8 M Urea with 50 mM ABC to make the solution suitable for trypsin digestion. The sample was digested overnight at 37 °C using a trypsin:protein weight ratio of 1:50, and the digestion was terminated by addition of 10% trifluoroacetic acid. The total digested peptides were desalted and concentrated with Macro Spin Column (C-18; Harvard Apparatus, Holliston, MA, USA) prior to high pH reversed-phase fractionation. The volume of the eluted peptide sample was evaporated in a vacuum centrifuge (SpeedVac) and reconstituted with 100 μL of 20 mM ammonium formate in water (pH 10.0).

High pH reversed-phase fractionation

The tryptic peptides from both sample sets of SPTD case and TD control groups were, respectively, separated by high pH reversed-phase fractionation using an 1260 HPLC infinity purification system (Agilent Technology, Santa Clara, CA) equipped with a Xbridge C-18 column (ZORBAX, 4.6 x 250 mm, 5 μm, 300 Å; Waters, Milford, MA) with the following buffers: buffer A (10 mM ammonium formate in water, pH 10.0) and buffer B (10mM ammonium formate in 90% acetonitrile, pH 10.0). The peptides were separated at a flow rate of 0.4 mL/min with gradient ramped from 0 to 15% of buffer B in 5.5 min, 15% to 28.5% in 24.5 min, 28.5% to 34% B in 4 min, 34% to 60% B in 10 min, 60% B for 4 min, then to 100% A in 4 min, and 100% A in 8 min. The collected peptide of 96 fractions were combined into 8 pools and desalted by Pierce C-18 spin column (Thermo Fisher Scientific, Bremen, Germany). The desalted peptide samples were lyophilized under vacuum (SpeedVac) and each of those fractions was then reconstituted with 0.1% formic acid in water prior to LC-MS/MS analysis.

LC-MS/MS analysis

Two μL of the tryptic peptide mixture was automatically loaded onto a precolumn (nanoViper Acclaim PepMap 100 C18, 75 μm x 2 cm, 3 μm and 100 Å pore size, Thermo Fisher Scientific, Bremen, Germany) at a flow rate of 3 μL/min. Then, the peptides were separated using a PepMap RSLC C-18 column (75 μm x 50 cm, 2 μm, 100 Å pore size particles; Thermo Fisher Scientific, Bremen, Germany) with a 69 min linear gradient increasing: the gradient elution started at 5% of mobile phase B (0.1% formic acid in

acetonitrile), followed by a 66 min gradient from 5% to 38% B, then increased to 90% of B and remained at this state for the 10 min at a constant flow rate of 200 nL/min. The eluted peptides from the column were directly electrosprayed into a Q-Exactive Mass Spectrometer. Fragmentation of the peptides was performed in data-dependent mode using a top 10 higher-energy collisional dissociation (HCD) MS/MS method (up to 10 HCD MS/MS spectra were acquired following each MS scan) operating in positive mode in the survey scan (300 to 1,600 m/z). The resolution of the survey scan was 70,000 with an automatic gain control of 1×10^6 ions, one microscan and maximum injection time of 50 ms. The maximum injection time for MS/MS was 120 ms. Dynamic exclusion was enabled 1 repeat count for 20 s after one MS/MS spectra acquisition. Samples from the SPTD case and TD control groups were analyzed by LC/MS as three technical triplicates.

Protein identification and label-free quantitative analysis

The SEQUEST search algorithm (Sorcerer v 4.3.0 built, Sage-N Research, Milpitas, CA) was used for searching each LC-MS/MS file against the UniProt protein database (42,083 entries; released in Mar 2015). Mass tolerance for peptides and MS/MS fragments were 10 ppm and 1 Da, respectively. A maximum number of two missed cleavages by trypsin was set for all searches. Cysteine carbamidomethylation (+57.021 Da) was set as a fixed modification, and methionine oxidation (+15.995 Da) was set as a variable modification. Scaffold 4 (version 4.3.2, Proteome Software Inc., Portland, OR) was used to filter peptide and protein identifications for an estimated false discovery rate (FDR) of less than 1%. Comparative analysis between corresponding sets of identified proteins was achieved by manual data

interpretation using Microsoft Excel, extracting statistical data such as number of distinct peptides, number of spectra, and sequence coverage. Three replicate LC-MS/MS runs from each group of sample were performed and the spectral counts of all identified proteins were $\log_2$-transformed and compared using the R statistical programming with a power law global error model (PLGEM, http://www.bioconductor.org) in order to identify proteins showing statistical significant changes (p-value of 0.05) between the SPTD case and TD control groups.[19-21]. The relative abundance ratios of proteins that exhibited statistically significant changes were calculated as ratios between spectral counts in the SPTD cases and those in TD controls, and the criterion $\log_2$ fold change (FC) > 0.3 (either up or downregulated) was applied for selecting differentially expressed proteins (DEPs) with statistically significant changes. Therefore, only the proteins that met these criteria were considered as significantly different for further analyses.

Ingenuity pathway analysis (IPA)

Taxonomy was set to human and, based on the published literature, the disease and canonical pathways connecting to all DEPs were visualized. Signaling networks were generated using the knowledge base for interactions between the uploaded DEP lists and all other stored gene objects. Functional analysis of the signaling networks was pursued to identify the biological functions and pathways that were most significant to the genes in the network.

Analysis of various proteins in the amniotic fluid

The ranges of CA-1, FCGBP, IGFBP-4, lipocalin-2, S100 A8, S100 A8/A9, VEGFR-1 standard curves were 78.125-5000 pg/mL, 0.78-50 ng/mL, 0.5-32 ng/mL, 78.1-5000 pg/mL, 31.25-2000 pg/mL, 93.8-6000 pg/mL, and 125-8000 pg/mL, respectively. Prior to measurement of these proteins, the AF samples were diluted at 1:1 for FCGBP, 1:2 for CA-1, 1:4 for S100 A8, 1:100 for IGFBP-4, and 1:500 for lipocalin-2, S100 A8/A9 and VEGFR-1.

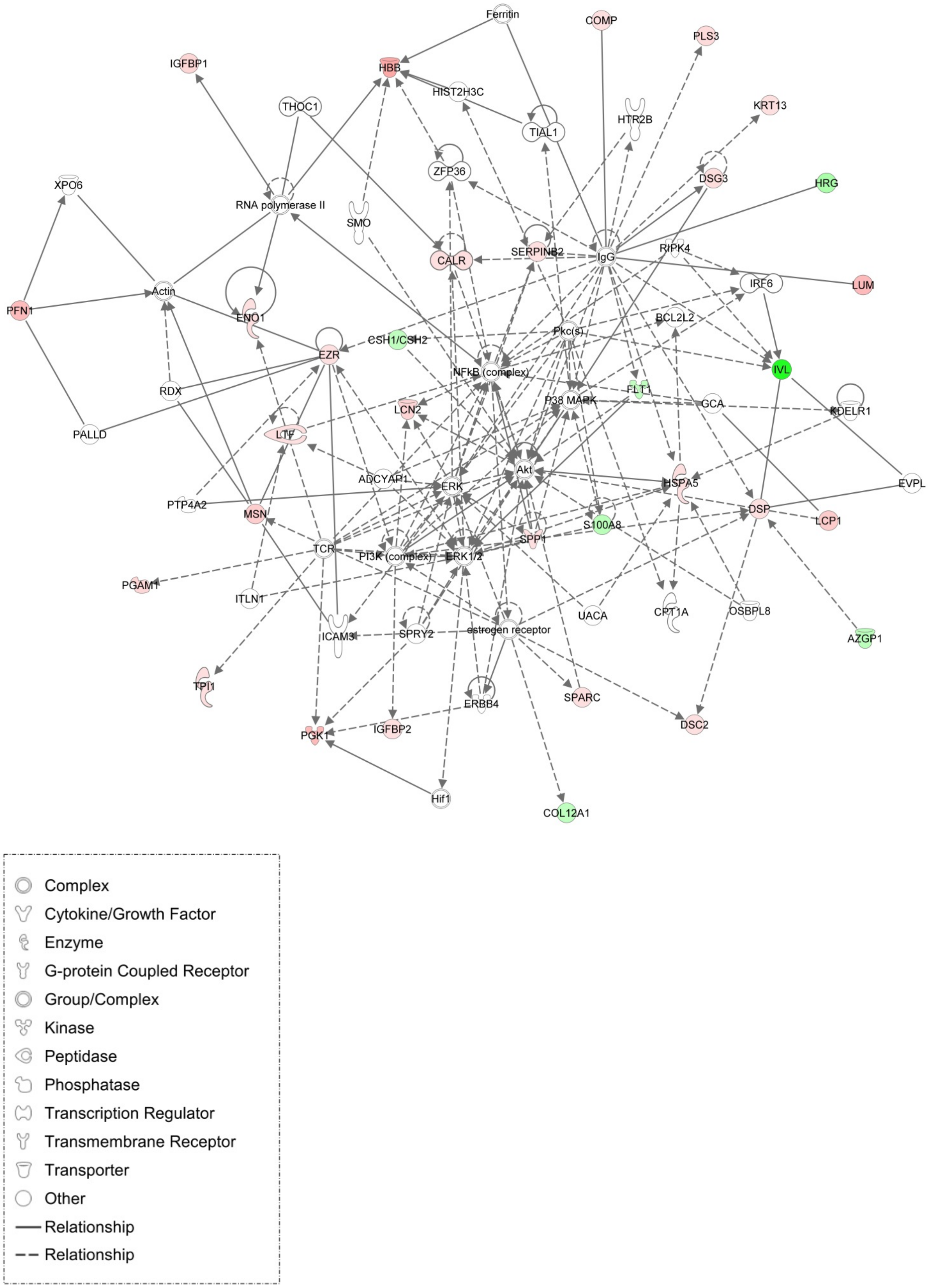
Ferritin
COMP
PLS3
IGFBP1
HBB
HIST2H3C
THOC1
TIAL1
HTR2B
KRT13
ZFP36
DSG3
HRG
XPO6
RNA polymerase II
SMO
CALR
SERPINB2
IgG
RIPK4
LUM
IRF6
PFN1
Actin
ENO1
BCL2L2
CSH1/CSH2
Pkc(s)
EZR
NFkB (complex)
IVL
RDX
LCN2
P38 MAPK
FLT1
GCA
KDELR1
PALLD
LTF
Akt
ADCYAP1
HSPA5
EVPL
ERK
PTP4A2
MSN
S100A8
DSP
LCP1
SPP1
TCR
PI3K (complex)
ERK1/2
PGAM1
ITLN1
UACA
CPT1A
OSBPL8
AZGP1
ICAM3
SPRY2
estrogen receptor
TPI1
ERBB4
SPARC
DSC2
PGK1
IGFBP2
Hif1
COL12A1
Complex
Cytokine/Growth Factor
Enzyme
G-protein Coupled Receptor
Group/Complex
Kinase
Peptidase
Phosphatase
Transcription Regulator
Transmembrane Receptor
Transporter
Other
Relationship
Relationship
