# Supplementary material for: Identifying potential biomarkers related to pre-term delivery by proteomic analysis of amniotic fluid
Source: Sci Rep. 2020 Nov 12;10:19648. doi: 10.1038/s41598-020-76748-1 (PMC7665029; doi:10.1038/s41598-020-76748-1)
Supplement: Supplementary file 3 — Supplementary Legend. [file 41598_2020_76748_MOESM3_ESM.docx]

**Supplementary Information**

**Figure S1.** Top interaction networks generated by ingenuity pathway analysis were merged to obtain a global view. The top network contained 32 proteins, including interacting molecules that were not found in this study. Proteins that were overexpressed in the SPTD case group are displayed in red, whereas proteins that were overexpressed in the TD control group are highlighted in green. NF-κB and P38 MAPK are seen as the major hubs in the network. Straight lines suggest direct interactions, whereas dashed lines represent indirect ones (The permission of QIAGEN Silicon Valley for the use of this figure is acknowledged, 2020).
